# Detecting relapse in youth with psychotic disorders utilizing patient-generated and patient-contributed digital data from Facebook

**DOI:** 10.1038/s41537-019-0085-9

**Published:** 2019-10-07

**Authors:** M. L. Birnbaum, S. K. Ernala, A. F. Rizvi, E. Arenare, A. R. Van Meter, M. De Choudhury, J. M. Kane

**Affiliations:** 1 The Zucker Hillside Hospital, Northwell Health, Glen Oaks, NY USA; 2 Feinstein Institute of Medical Research, Manhasset, NY USA; 3 Hofstra Northwell School of Medicine, Hempstead, NY USA; 4 Georgia Institute of Technology, Atlanta, GA USA

**Keywords:** Psychosis, Human behaviour

## Abstract

Although most patients who experience a first-episode of psychosis achieve remission of positive psychotic symptoms, relapse is common. Existing relapse evaluation strategies are limited by their reliance on direct and timely contact with professionals, and accurate reporting of symptoms. A method by which to objectively identify early relapse warning signs could facilitate swift intervention. We collected 52,815 Facebook posts across 51 participants with recent onset psychosis (mean age = 23.96 years; 70.58% male) and applied anomaly detection to explore linguistic and behavioral changes associated with psychotic relapse. We built a one-class classification model that makes patient-specific personalized predictions on risk to relapse. Significant differences were identified in the words posted to Facebook in the month preceding a relapse hospitalization compared to periods of relative health, including increased usage of words belonging to the swear (*p* < 0.0001, Wilcoxon signed rank test), anger (*p* < 0.001), and death (*p* < 0.0001) categories, decreased usage of words belonging to work (*p* = 0.00579), friends (*p* < 0.0001), and health (*p* < 0.0001) categories, as well as a significantly increased use of first (*p* < 0.0001) and second-person (*p*  < 0.001) pronouns. We additionally observed a significant increase in co-tagging (*p* < 0.001) and friending (*p* < 0.0001) behaviors in the month before a relapse hospitalization. Our classifier achieved a specificity of 0.71 in predicting relapse. Results indicate that social media activity captures objective linguistic and behavioral markers of psychotic relapse in young individuals with recent onset psychosis. Machine-learning models were capable of making personalized predictions of imminent relapse hospitalizations at the patient-specific level.

## Introduction

Schizophrenia and other psychotic disorders can be associated with significant impairment.^1^ Although the majority of patients with first-episode psychosis initially achieve clinical remission of hallucinations and delusions, up to 80% experience at least one relapse within the first 5 years.^2^ Each new episode can be associated with costly emergency room visits, psychiatric hospitalizations, family burden, medical complications, legal issues, and suicide.^3,4^

There is substantial evidence, suggesting that psychotic symptom exacerbation is preceded by periods of anxiety, low mood, sleep pattern irregularity, trouble concentrating, social withdrawal, strained interactions with others, altered psychomotor activity, and attenuated psychotic symptoms.^5,6^ Clinical interview, patient self-report, and family observation remain the primary sources for gathering early warning signs.^7,8^ Unfortunately, the utility of these strategies is severely limited by the need for direct, frequent, and timely contact with trained professionals, as well as accurate and insightful patient and family recall. Continuous, objective monitoring of burgeoning psychotic symptoms could facilitate the initiation of early and proactive relapse prevention strategies.^9,10^

The dramatic rise in social media use could provide an opportunity to inform early relapse identification. Social media platforms have transformed the ways by which people interact, communicate, and share information. The majority of U.S. teens and young adults use social media every day^11,12^ and many report a tendency to disclose more sensitive information about themselves online than in-person.^13^ Similarly, youth with psychotic disorders reported regularly utilizing social networking sites, engaging in social media activity several times daily and spending nearly 2 hours per day online.^14,15^

Harvesting social media data has become an established method for capturing health information about an individual or a population through explicit commentary, patterns and frequency of use, as well as the intricacies of language.^16–19^ Social media sites, like Facebook and Twitter, store data as time-stamped digital records, providing a detailed source of collateral information about an individual’s experience and behavior over an extended period of time. Interest has grown in the potential for social media activity to be used in behavioral health as a tool to assist in diagnosing and monitoring patients receiving psychiatric care.^20^ Patterns of social media use have been used to predict demographic attributes, personality traits, intelligence, happiness, substance use, and subjective well-being with high degrees of accuracy.^21–24^ Language extracted from Facebook status updates has been shown to be associated with symptoms of depression in college students^25^ and individuals presenting to the Emergency Department.^26^ Linguistic changes in Twitter posts have been linked to the onset of a depressive episode^27,28^ and the emergence of suicidal ideation.^29,30^ Furthermore, there is compelling evidence, suggesting that subtle changes manifest in social media activity before they become clinically apparent, providing the potential for earlier identification and intervention. Changes in social media-based linguistic and behavioral activity, for example, have been shown to reliably predict future episodes of depression,^27^ postpartum mood disorders,^31^ binge drinking behavior,^32^ and self-disclosures of schizophrenia^33,34^ with high degrees of accuracy.

Although promising, this line of research is limited by the fact that it has been conducted primarily using publicly available social media data, has relied largely on anonymous self-disclosed or self-reported diagnoses of mental illness, and has rarely been validated for its theoretical and clinical grounding and validity.^35^ Importantly, in order to make clinical use of social media data, it is crucial that these initiatives include collaborations with mental health clinicians, using data from known patients with confirmed diagnoses. While the limits of predictive models trained on public data from the perspective of construct validity and population bias have been raised,^35,36^ there are currently few studies that combine the expertize of both computer scientists and mental health professionals to assess the generalizability and robustness of these data and machine-learning models built on them, in clinical contexts. Although some self-disclosed diagnoses of “depression” or “schizophrenia” may be accurate, and survey based self-reports correct, it is challenging to confirm their authenticity, and it is clear that these labels are often used incorrectly.^33^ Investigators analyzing public data do not have access to a reliable way of validating patient groups. Consequently, little is known about how well the resulting models would perform in individuals with diagnosed mental health disorders. Additionally, although there are defined criteria for diagnosing mental illness,^37^ there is substantial heterogeneity in symptom presentation and functioning within a diagnostic category,^38^ and making accurate diagnoses takes training and clinical experience. In order to develop models with true clinical utility as part of the diagnostic and treatment process, real patient data must be used.

We sought to conduct an ecologically valid investigations into the relationship between social media activity and behavioral health. Specifically, we aimed to identify and predict early relapse warning signs in social media activity collected from a cohort of individuals receiving psychiatric care for schizophrenia and other primary psychotic disorders. To achieve this goal, we tested a machine-learning model to predict relapse events by differentiating temporal periods preceding hospitalizations for symptomatic exacerbations from periods of relative health. The model leverages patient Facebook data and dates of hospitalizations from their medical record, and was designed to make predictions at an individual level, consistent with a personalized approach to medicine.^39^ We generated features from Facebook timeline data grounded in the symptomatic and functional impairments associated with psychotic disorders.^40^ These include: (1) word usage and psycholinguistic attributes related to affective, social, and personal experiences,^41,42^ (2) linguistic structural attributes, such as complexity, readability, and repeatability related to thought organization and cognitive abilities,^43^ and (3) online activities relating to social functioning and diurnal patterns,^44^ such as friending, posting, and check-ins. We hypothesized that psycholinguistic attributes, linguistic structure, and patterns and timing of data posted to Facebook during periods preceding a relapse hospitalization would be distinct from data posted during periods of relative health. Additionally, we expected differences in these aspects of Facebook posts to grow larger, consistent with symptom exacerbation, closer to the date of hospitalization.

A key challenge in predicting relapse hospitalizations is the relative rarity of these events compared to periods of health, causing a class imbalance when binary classification approaches are adopted. Further, while most periods of relative health are similar, each relapse hospitalization can be unique, even within the same individual.^45,46^ To handle the skewed distribution of classes (periods of relapse and relative health) and the heterogeneity within the rare class, we adopted supervised anomaly detection techniques—specifically one-class classification algorithms for prediction,^47,48^ which distinguishes between “normal” and “anomalous” observations.^49^ This methodological framework can enable efficient intervention by predicting anomalies or exacerbations indicative of relapse in a personalized manner based on learned patterns of behaviors during healthy periods.

We compiled hospitalization dates and Facebook archives from 110 consenting participants with a psychotic disorder. Of those, 37 were excluded as they did not have a relapse hospitalization, 14 had missing hospitalization dates, 3 had never been hospitalized, and 5 had unusable archive data (2 had insufficient Facebook data, 1 had primarily non-English data, 2 were unable to parse). Using the hospitalization dates as markers, each participant’s Facebook data was segmented into periods of relapse and periods of relative health. The one-class classification algorithm was then trained on periods of relative health to identify distinguishing patterns of inliers. The best performing model was then tested on an unseen sample of both periods of relapse and relative health with the goal of predicting healthy periods as inliers and relapse periods as outliers. We assessed the validity of the model on patient-specific predictions based on the inferential ability (specificity, sensitivity). Finally, we conducted an error analysis by accessing data from medical records to understand the specific instances of mislabeled data or incorrect predictions by the model.

## Results

### Data description

A total of 52,815 Facebook posts (mean = 71.08, SD = 366.78) were collected across 51 participants (mean = 71.08, SD = 366.78) who had been diagnosed with a primary psychotic disorder (mean age = 23.96 years; 70.58% male) and had at least one relapse hospitalization (Table 1). There was an average of 2.4 relapse hospitalizations per participant with a median hospitalization stay of 13 days.

**Table 1:** Participant demographics

Participant Demographics	Mean (years)	SD
Age	23.96	4.59
Sex	**N**	**%**
*Male*	36	70.58
*Female*	15	29.41
Race		
*Asian*	5	9.80
*African American*	28	54.90
*Caucasian*	11	21.56
*Other/ Mixed*	7	13.72
Diagnosis		
*Schizophrenia*	34	66.66
*Schizoaffective Disorder*	13	25.49
*Unspecified Schizophrenia Spectrum Disorder*	4	7.84

### Exploratory analysis

Comparing linguistic and behavioral features during periods of relative health to periods of relapse, randomly sampled per participant, identified significant differences across several categories (Table 2). We observed increased usage of words belonging to the anger (*p* < 0.001, Wilcoxon signed rank test), death (*p* < 0.0001), swear (*p* < 0.0001), negative affect (*p* < 0.001), hear (*p* < 0.0001), and feel (*p* < 0.01) categories during periods preceding a relapse hospitalization. We also observed an increased usage of pronouns during the period preceding a relapse hospitalization, including first-person plural (*p* < 0.0001) and second-person (*p* < 0.01) compared to periods of relative health. Among the social media activity-based features, we observed an increase in co-tagging (*p* < 0.001) and friending (*p* < 0.0001) behaviors, as well as heightened posting activity between 05:00 a.m. and 12:00 p.m. (*p* < 0.01) and between 22:00 p.m. and 05:00 a.m. (*p* < 0.01) prior to a relapse hospitalization. Additionally, we observed significantly decreased use of words belonging to the work (*p* < 0.01), achievement (*p* < 0.05), friends (*p* < 0.0001), body (*p* < 0.01), and health (*p* < 0.0001) categories during periods of relapse.

**Table 2:** Wilcoxon-signed rank test results comparing linguistic differences between a period of relapse and period of relative health per participant

Feature	Mean during periods of relapse	Mean during periods of relative health	*t*-statistic	*p*-value
Co-tagging noon	0.4074	0.1481	18.0	<0.0001
Posting morning	21.8518	4.1481	74.5	<0.01
Co-tagging	2.5185	0.7777	44.0	<0.001
Co-tagging midnight	0.8148	0.2222	13.5	<0.0001
Co-tagging morning	0.9630	0.2222	5.5	<0.0001
Friending	0.2593	0.2222	2.5	<0.0001
Co-tagging night	0.3333	0.1851	4.0	<0.0001
Posting midnight	12.1481	4.4814	44.5	<0.01
Exclusive	0.0063	0.0079	92.0	<0.05
Family	0.0028	0.0021	18.0	<0.0001
Inclusive	0.0253	0.0152	101.0	<0.05
Feel	0.0059	0.0057	59.0	<0.01
Money	0.0022	0.0020	18.0	<0.0001
Causation	0.0028	0.0113	37.0	<0.001
Insight	0.0073	0.0076	69.0	<0.01
Humans	0.0044	0.0043	66.0	<0.01
Anger	0.0049	0.0032	36.5	<0.001
Home	0.0019	0.0045	28.5	<0.001
Sexual	0.0032	0.0033	30.0	<0.001
Future tense	0.0036	0.0016	10.0	<0.0001
Death	0.0008	0.0004	16.5	<0.0001
Negation	0.0075	0.0091	98.0	<0.05
Discrepancies	0.0064	0.0051	41.0	<0.001
Religion	0.0029	0.0025	29.0	<0.001
Verbs	0.0635	0.0856	104.0	<0.05
Health	0.0023	0.0092	11.0	<0.0001
First-person plural	0.0016	0.0005	8.0	<0.0001
Bio	0.0098	0.0180	78.0	<0.01
Tentativeness	0.0070	0.0067	93.5	<0.05
Body	0.0041	0.0045	54.5	<0.01
Inhibition	0.0023	0.0012	12.0	<0.0001
Hear	0.0066	0.0024	25.0	<0.0001
Second-person	0.0081	0.0070	71.0	<0.01
Quantifier	0.0052	0.0028	55.0	<0.01
Friends	0.0042	0.0071	26.0	<0.0001
Achievement	0.0061	0.0067	85.0	<0.05
Negative affect	0.0029	0.0023	42.5	<0.001
Anxiety	0.00078	0.0008	9.0	<0.0001
Certainty	0.0040	0.0091	51.0	<0.001
Work	0.0082	0.0164	74.5	<0.01
Indefinite pronoun	0.0151	0.0235	91.0	<0.05
Sadness	0.0016	0.0019	19.0	<0.0001
Swear	0.0034	0.0017	16.0	<0.0001

### Machine-learning model to predict relapse events

We built three one-class support vector machine (SVM) models^48^ for three different data configurations: (1) periods of relapse and periods of relative health as 1-month temporal periods (1-month model), (2) periods of relapse as 1-month temporal periods and periods of relative health as 2-month periods (2-month model), (3) period of relapse as 1-month temporal periods and periods of relative health as 3-month periods (3-month model). A 1-month relapse period was selected as it represents a period of time prior to hospitalization during which early relapse warning signs typically become clinically apparent.^50–52^

Each one-class SVM model is trained on temporal periods of relative health as inliers (positive class) and then tested on an unseen sample of both periods of relapse (outliers/negative class) and relative health (Table 3). We then compared the performance of all three models based on their sensitivity and specificity (Table 4). We found that the 1-month model had the highest specificity of 0.65 when compared to the 2-month or 3-month model (specificity of 0.28 and 0.04, respectively). This affirmed our expectation that behaviors characteristic to relapse would be most dominant during the 1-month period preceding a relapse (closer to the hospitalization). On the other hand, we found that the 1-month model performed worst in correctly predicting the healthy periods (sensitivity of 0.47) when compared to the 2-month or 3-month model (sensitivity of 0.57 and 0.90, respectively). This trend shows the trade-off between availability or volume of data and predictive performance revealing that incorporating longer periods of relative health (higher volume of data) helps in correctly predicting healthy periods but the performance on relapse prediction worsens. Given that the goal of this initiative was to predict relapse, and the clinical value in identifying symptomatic exacerbations, we emphasized the significance of specificity over sensitivity.

**Table 3:** Descriptive statistics on Facebook timeline data comprising participant generated posts

Data configuration	# Periods of relapse	# Periods of relative health	Total number of posts (periods of relapse)	Total number of posts (periods of relative health)	Mean, SD of number of posts (periods of relapse)	Mean, SD of number of posts (periods of relative health)
1-Month periods of relapse and relative health	49	719	1708	51,107	34.86, 136.25	71.08, 366.78
1-Month periods of relapse and 2-month periods of relative health	49	421	1708	50,161	34.86, 136.25	119.15, 640.52
1-Month periods of relapse and 3-month periods of relative health	49	312	1708	50,108	34.86, 136.25	160.60, 911.37

**Table 4:** Class distributions and model performance on unseen test data for the one-class SVM models

	# Periods of relative health	# Periods of relapse	Sensitivity	Specificity	Positive predictive value	Negative predictive value
1-Month model	719	49	0.47	0.65	0.66	0.46
2-Month model	419	49	0.57	0.28	0.41	0.44
3-Month model	312	49	0.90	0.04	0.37	0.4
Ensemble model	719	49	0.38	0.71	0.66	0.44

In order to improve the performance of the 1-month model, we built an ensemble one-class support vector machine algorithm (details in Supplement). Ensemble methods are algorithms that combine multiple machine-learning models into one to reduce errors and decrease variance in predictions. The ensemble model was trained on 90% of 1-month periods of relative health as inliers and was tested on an unseen sample of 10% 1-month periods of relative health and all of the periods of relapse. The model predicts whether a given time period will have an adverse outcome such as relapse hospitalization. This ensemble model correctly predicted unseen relapse periods as outliers with a specificity of 0.71 and sensitivity of 0.38. Table 4 provides further details on the model performance. With the emphasis on specificity, we find that the ensemble model performs better than the individual models in predicting periods of relapse with the highest specificity. However, the performance lowered in terms identifying periods of relative health.

### Error analysis: evaluation via clinical chart review

Given that the goal of the classifier is to predict periods of relapse, we conducted a deeper analysis of the misclassifications made by the model, specifically false negatives (periods of relative health wrongly predicted as a relapse). Note that the models consider periods of health as positive (inliers) and periods of relapse as negative examples (outliers). For each misclassified time period, two co-authors reviewed the accompanying clinical records. For 20 out of the 45 false-negative time periods (44%), data was available from the patient’s medical record. In 18 of these 20 instances, the presence of psychotic symptoms during periods defined as relative health was documented, and six of these participants had known non-adherence to medication during this time which can contribute to symptomatic exacerbations.^2^ Thus, of 20 periods for which symptom status could be verified from the medical record, 18 represented periods during which there was significant psychotic symptom exacerbation, even though the severity threshold necessitating hospitalization was not reached. There were also five instances that were incorrectly predicted by the model to be periods of relapse (false positives); however, a relapse hospitalization did indeed occur within the subsequent 2-month window or the participant was admitted into an intensive day treatment program. These periods may therefore represent *true* periods of relapse.

## Discussion

This research aimed to identify early psychosis relapse warning signs from linguistic and behavioral features extracted from Facebook. It utilized social media data from patients diagnosed with a psychotic disorder by a mental health professional, and incorporated behavioral health data from medical records. We believe this is a significant step toward the goal of leveraging social media activity to improve mental health services.^26,27,53,54^ Further, this work allows us to go beyond utilizing social media activity to identify population-based, or group-level characteristics, associated with mental health status—nearly exclusively the only approach employed in prior research. With our machine-learning approach, we have demonstrated that personalized methods to longitudinally forecast the likelihood of imminent adverse mental health outcomes, like a relapse event, is feasible. Specifically, we identified significant changes in Facebook activity in the month preceding a relapse hospitalization for psychosis, and built an individual-centric classifier achieving a specificity of 0.71 in predicting psychotic relapse using both linguistic and behavioral data.

Prior research in linguistic analysis has identified significant differences cross-sectionally at the word level in the use of certain word categories,^55–62^ as well as at the sentence level in terms of semantic density, coherence, and/or content,^60–70^ both in individuals at risk for developing psychotic disorders, as well as those with established schizophrenia spectrum disorders. The majority of studies to date have extracted linguistic data from speech or written text providing large volumes of analyzable sentences. This work contributes by examining and identifying changes in language used in social media posts associated with symptom escalation among individuals diagnosed with a psychotic disorder. Social media has emerged as an increasingly dominant source of language data, especially among adolescents and young adults.^11,12^ Facebook status updates and social media-based communication, however, is unique due to short sentence structure, abbreviations, and distinct writing styles, and requires careful consideration when developing and adopting language-based algorithms to predict mental health status.

We identified significantly increased use of words belonging to the swear, anger, and negative emotion categories in the period of time preceding a relapse hospitalization consistent with escalating irritability and depression known to be associated with emerging relapse.^50,71^ We also found increased use of words belonging to the hear and feel categories in the month preceding a relapse hospitalization, consistent with emerging perceptual disturbances, commonly experienced by individuals with psychosis.^50,51,71^ This is also consistent with prior work in those at risk for developing psychosis, suggesting that words related to auditory perception, such as voices and sounds, predicted conversion to psychosis.^62^ Consistent with prior studies, we found increased use of first-person pronouns,^59,63^ but also second-person pronouns, which may be indicative of changes in the way an individual thinks about him/herself in relation to others, in-line with the social changes prominent in individuals experiencing worsening symptoms of psychosis.^50,51,71^ Increased use of first-person pronouns may also be indicative of emerging self-referential thinking, a common psychotic experience contributing to delusions, whereby neutral environmental stimuli are perceived to be personally meaningful.^37^ In contrast to those at risk for developing psychotic disorders, we did not find that the use of determiners^60^ or possessive pronouns^61^ were associated with psychotic symptom exacerbation, which may represent linguistic differences among individuals who develop a psychotic disorder compared to those who relapse, or more likely, differences in the way individuals communicate through speech versus Facebook. Finally, we observed decreased use of words relating to work, achievement, friends, and health consistent with declining academic functioning and social isolation often associated with psychotic relapse.^50,51,71^

An important next step will involve determining which linguistic features (or combination of features) are specific to psychotic relapse rather than an indication of worsening mental health status. For example, prior research has similarly identified increased self-referential language, as well as words related to negative emotions in individuals with depressive disorders and suicidal thoughts and actions.^29,30,59,72,73^ This question will need to be explored in future initiatives comparing social media data from individuals with symptomatic exacerbations across multiple diagnostic groups to assess specificity for psychosis relapse.

In addition to linguistic changes, social media activity offers digital representations of potentially clinically meaningful behavioral patterns associated with psychotic disorders and incipient relapse. We identified a significant increase in co-tagging and friending behaviors in the month preceding a relapse hospitalization, as well as increased posting activity after midnight and into the early morning. While social dysfunction is a hallmark of schizophrenia,^37^ precisely how offline social behaviors manifest through social media has yet to be determined. Increased co-tagging and friending activity prior to a relapse event may represent inappropriate and/or disorganized social behavior often seen in individuals with worsening psychosis.^50,51,71^

We additionally identified several features that proved critical to our relapse classifier, including the total amount of friending, tagging, photo uploads, reposts, and likes, as well as nighttime posting, and information sharing in the late evening and very-early morning. These features most likely represent digital representations of behavioral changes associated with escalating psychotic symptoms, including disruptions in sleep and circadian rhythm, disturbances in social functioning, and shifting interests and activities.^50,51,71^ Other initiatives have supported the use of technology to augment relapse prediction for individuals with mental illness, including schizophrenia. Most research to date has focused on the association between objectively recorded smartphone sensor data, including geolocation, physical activity, phone usage, and speech and clinical state or symptom fluctuations.^52,74–76^ Our results demonstrate that user-generated social media activity represents an equally critical source of digital data contributing to relapse identification. Future work combining digital data from multiple sources will likely result in the most effective clinical tools.

Combining linguistic and behavioral features resulted in a classifier that predicted relapse with an accuracy of 71%, however, low sensitivity (0.38) limits the clinical utility of our model. Performance was likely impacted by our definition of relapse, which was defined as a hospitalization due to psychotic symptoms. Relapse, however is a complicated phenomenon, and has other definitions, including symptomatic exacerbations that do not result in hospitalization.^77^ Furthermore, the decision to hospitalize is often multifactorial and may not always be a reliable indicator of psychotic symptoms. Our error analysis suggested that several periods believed to be incorrectly identified as periods of relapse did in fact have documented evidence for the presence of psychotic symptoms, although they did not necessarily result in a hospitalization. As we continue to explore digital manifestations of psychotic symptom exacerbation, researchers will need to identify models that have both high specificity and high sensitivity in predicting relapse. To be clinically useful, models will need to be capable of accurately predicting emerging relapse while avoiding false positives that would unnecessarily increase clinician burden and could negatively impact patient outcomes. False negatives could also be detrimental, particularly if clinicians relied on model prediction and failed to intervene in spite of concerning clinical changes.

There are several noteworthy limitations. In addition to the definition of relapse described above, our approach was limited by our characterization of monthly periods of relative health and relative illness. Illness trajectory for many individuals with psychotic disorders does not neatly fall into distinct segments of “health” and “illness,” rather symptoms fluctuate over time. Furthermore, the recording of inpatient hospitalization dates were obtained via medical records, and it is possible that some hospitalizations were missing from the record and, therefore, not included in our analyses. In order to address these limitations and to improve our ability to find associations between social media activity and psychotic symptom exacerbations, future studies need to monitor participants prospectively and utilize frequent symptom rating scales to more accurately assess symptom severity. Secondly, while all participants included in our analyses experienced at least one relapse hospitalization, the specific symptoms that define an exacerbation for each individual with psychotic disorders are often unique, and although symptom heterogeneity was addressed in our analyses, generalizability may be limited. Third, some participants were more active on Facebook than others, providing varying degrees of extractable data. An important question for future research will be how much social media data is necessary in order to make a reliable clinical predictions. Fourth, the Facebook archives used for our analyses were collected retrospectively. While retrospective collection eliminates the possibility of altering behavior as a result of being monitored, to achieve the goal of early relapse identification, prospective monitoring will be necessary in future work. Finally, eligibility criteria ranged from 15 to 35 years to reflect the inclusion criteria of the Early Treatment Program, however, adolescents may engage with social media in a distinct manner compared to young adults and will need to be considered in future initiatives aimed at predicting relapse events.

Social media platforms hold promise for gathering objective, non-invasive, and easily accessed, indicators of psychotic relapse. This knowledge represents advancement in efforts to capitalize on objective digital data to improve mental health monitoring, and supports the development of a new generation of innovative and targeted clinical tools by employing social media-based language and behavior analysis. Going forward, integrating multiple sources of digital data (sensors, social media, online searches) to predict mental health outcomes in clinical settings, could change the way clinicians diagnose and monitor patients, enabling faster, more accurate identification of symptom exacerbation and facilitating a personalized approach to medicine. This would be a significant step forward for psychiatry, which is limited by its reliance on largely retrospective, self-reported data. However, how these data and inferences are integrated into the existing clinical workflow and practice is an open question of inquiry and an important area of research. Interdisciplinary teams of researchers, clinicians, and patients must continue to work together on identifying and solving ongoing questions and challenges in ethics, privacy, consent, and clinical responsibility.^36,78^ The data utilized in the current study were obtained from consenting participants who were fully informed of the risks and benefits of participation. However, the potential for this type of information to reveal sensitive clinical insights may motivate other parties to collect and analyze it without consent. Importantly, investigators must develop standards to protect the confidentiality and the rights of this sensitive population to avoid misuse of personal information as our analyses become increasingly sophisticated and our ability to predict health information improves. Investigators must ensure that the data and the technologies are used in the service of positive outcomes for clinicians and the patients they treat.

## Methods

Participants between the ages of 15 and 35 years old who had been diagnosed with a primary psychotic disorder screened for eligibility from Northwell Health’s inpatient and outpatient psychiatric departments. Most were recruited from the Early Treatment Program (ETP), Northwell Health’s specialized early psychosis intervention clinic. Additional participants (*N* = 7) were recruited from three collaborating early psychosis programs located in Florida and Michigan (East Lansing and Grand Rapids). Individuals with secondary psychiatric comorbidities were included. Eligible participants were approached by a local research staff member and offered the opportunity to participate. Recruitment occurred between March 2016 and December 2018. The study was approved by the Institutional Review Board (IRB) of Northwell Health (the coordinating institution), as well as local IRBs at participating sites. Written informed consent was obtained locally for adult participants and legal guardians of participants under 18 years of age. Assent was obtained for participating minors. None of the participants were involved in intervention research and all were receiving treatment as usual.

All participants were asked to extract their Facebook archive by logging on to their Facebook account and requesting their history accessible in their settings. Participation involved a single visit at the time of consent during which all historical social media data was downloaded and collected. Archives include all uploaded content (comments, messages, shares, likes, photos, etc.) since account creation. All user-generated social media content and activity was available for analyses. Clinical data including dates of hospitalizations and diagnoses were obtained through medical records.

### Data description

**Fig. 1:**
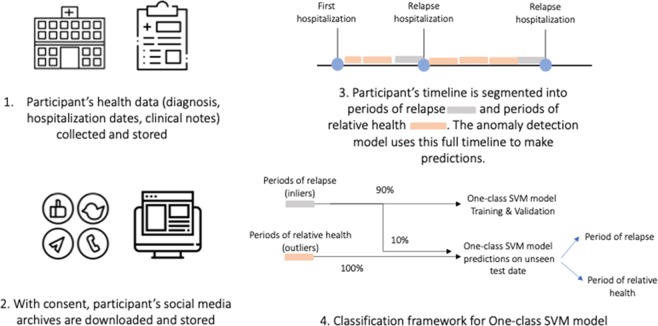
Flowchart of the relapse prediction machine-learning methodology

**Fig. 2:**
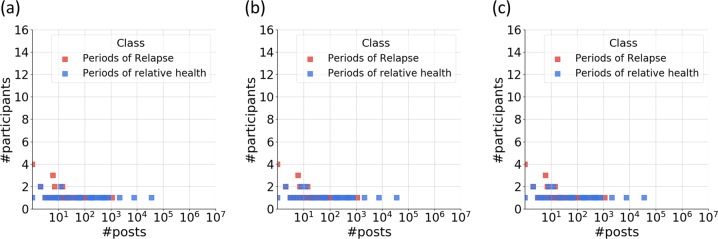
Distributions of number of participants, and number of posts for **a** 1-month, **b** 2-month, and **c** 3-month model

Each participant’s Facebook timeline data comprising self-generated posts from the day of the first hospitalization to the day of most recent hospitalization for a relapse was segmented into temporal periods (Fig. 1). Using the hospitalization dates per participant as markers, temporal periods 1 month prior to a relapse hospitalization were labeled as periods of relapse, as we expected to see symptom exacerbation most distinctive closer to the hospitalization. Excluding the 1-month preceding a relapse hospitalization, all other time periods were considered periods of relative health and representative of a person’s baseline behavior. Healthy periods were segmented at varying granularity ranging from 1 to 3 months to understand the tradeoffs between availability of data and performance of the model (Fig. 2).

### Classification framework

We built three models based on each of the data configurations described above: 1-month model, 2-month model, and 3-month model.

### Preparing training data

For the 1-month model, inliers correspond to 1-month temporal periods of relative health (*n* = 719) and outliers correspond to 1-month periods of relapses (*n* = 49). For the 2-month model, inliers comprises 2-month temporal periods of relative health (*n* = 421) and outliers comprises 1-month periods of relapse (*n* = 49). Finally, for the 3-month model, inliers comprises 3-month temporal periods of relative health (*n* = 312) and outliers comprises the same 1-month periods of relapse (*n* = 49) (Table 2). The training data used for the three models (1-month, 2-month, and 3-month) overlapped.

### Preparing unseen test data

Each of the three models was trained on 90% of the inliers and the remaining 10% of inliers alongside 100% of the outliers were held out as unseen data to test the classifier. Therefore, the held out data for the 1-month, 2-month, and 3-month model comprises 72, 42, and 31 periods of relative health, and 49 periods of relapse.

### Features

**Fig. 3:**
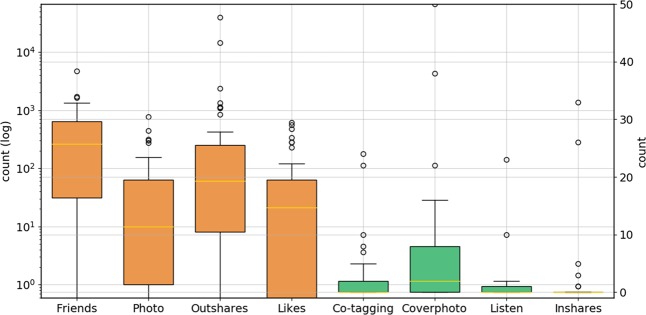
Descriptive statistics on Facebook activity-based features. Orange box plots correspond to the left-hand side *y*-axis and green box plots correspond to right-hand side *y*-axis

We used linguistic features such as word usage (through an n-gram language model) and psycholinguistic attributes (via LIWC)^41^ as a rich body of literature has identified associations of these attributes to emotion and behavior, including mental health states.^41,42^ To capture structural aspects of language in social media, we used linguistic readability, word repeatability, and word length as features to the model (details in Supplement). To capture behavioral measures on social media, providing insight into social functioning, diurnal patterns, sleep, and interests, we extracted volume and timing of posts, and Facebook activities such as check-ins, co-tagging, liking, sharing content, and using third-party apps (Fig. 3).

### Feature selection

Combining all of the attributes transformed the data into 607 features. We applied a feature selection method based on the coefficient of variance^79^ that quantifies the ratio of standard deviation to the mean value for each feature as a measure of dispersion. We eliminated features that had a coefficient of variance one standard deviation away from the mean and filtered a final set of 79 features (details in Supplement).

### Reporting summary

Further information on research design is available in the Nature Research Reporting Summary linked to this article.

## Supplementary information

Supplemental Information

Reporting Summary

## Data Availability

The datasets analyzed during the current study are not publicly available due to participant privacy and security concerns, including HIPAA regulations. The Facebook archives and health records are not redistributable to researchers other than those engaged in the IRB approved research collaborations from the author upon reasonable request.

## Code availability

The code implemented for the current study is not publically available due to participant privacy and security concerns, including HIPAA regulations.
