# Supplementary material for: Detecting relapse in youth with psychotic disorders utilizing patient-generated and patient-contributed digital data from Facebook
Source: NPJ Schizophr. 2019 Oct 7;5:17. doi: 10.1038/s41537-019-0085-9 (PMC6779748; doi:10.1038/s41537-019-0085-9)
Supplement: Supplementary file 1 — Supplemental Information [file 41537_2019_85_MOESM1_ESM.pdf]

Data description:
We extracted Facebook timeline data comprising 52,815 Facebook posts across 51 participants beginning from the first hospitalization for psychosis until the most recent hospitalization for a psychotic relapse. The total number of relapse hospitalizations across all participants was 124 with a mean of 2.4 relapse hospitalizations per participant. Participants spent an average of 17.7 days in the hospital per relapse hospitalization (SD=16.16, median=13). To avoid mislabeling periods during a hospitalization as either periods of relapse (preceding a hospitalization) or periods of relative health, we took the median of 13 days after every admission date and discarded the timeline data during this time.

Machine learning methodology:
Features**:** Here we describe the features from Facebook timeline data that were incorporated by the machine learning methodology. The first category of features included linguistic word usage computed using an n-gram language model. Each post was represented as a feature vector of normalized term frequency-inverse document frequency (tf-idf) counts of the top 500 *n*-grams. The second category of features included psycholinguistic attributes based on the well-validated Linguistic Inquiry and Word Count (LIWC) dictionary. We extracted attributes belonging to the following categories: 1. Affective attributes comprising positive and negative affect, anger, anxiety, sadness, and swear categories; 2. Cognitive attributes comprising cognitive mechanisms, discrepancies, inhibition, negation, causation, certainty, tentativeness, see, hear, feel, percept, insight, and relative categories; 3. Linguistic style attributes comprising function words (verbs, auxiliary verbs, adverbs, prepositions, conjunctions, articles, inclusive, and exclusive), temporal references (past, present and future tense), social and personal concerns (family, friends, social, work, health, humans, religion, bio, body, money, achievement, home, sexual, and death) and pronoun usage comprising 1st person singular, 1st person plural, 2nd person, and 3rd person pronouns. Each post was represented as a vector of normalized LIWC scores for each of the preceding 50 categories. The third category of features we computed related to linguistic structural measures such as readability, word repeatability, and word length. Readability was measured using the Coleman-Liau index[1] (CLI). CLI is a readability assessment test based on character and word structure within a sentence and approximates to a U.S. grade level required to understand the text and is calculated using the formula: $CLI = 0.0588L - 0.296S - 15.8$, where, L is the average number of letters per 100 words of content and S is the average number of sentences per 100 words. Word length and repeatability was measured as the normalized length of a word and normalized count of non-unique words in a post respectively. We considered Latent Dirichlet Allocation based topics as features to the model but qualitative evaluation revealed noisy topics. To restrict model complexity, we did not incorporate these noisy features. The final category of features included activity features from Facebook that were computed through regular expression matching from Facebook timeline posts. We identified the following activities on Facebook: adding new friendships, check-ins, co-tagging, updating photos or cover photos, sharing posts to others (out-shares) or others' sharing posts on a participant's timeline (in-shares), listening to music or playing third-party apps on the platform, liking, and adding new information to profile fields. Each post was represented as a feature vector of normalized counts of regular expression matches to Facebook activity updates. Additionally, the activity features were related to diurnal patterns by computing the normalized counts of activities during four epochs: morning, noon, night, midnight defined between 05:00-12:00, 12:00-17:00, 17:00-22:00, and 22:00-05:00 respectively.

All feature values were computed per post with normalization based on post length. Multiple posts made by an individual on the same day were aggregated by taking the average feature value per day across all posts.

Feature selection: We applied a coefficient of variance based feature selection method to eliminate noisy features and identify the most salient ones to predict the outcome. Coefficient of variance (cv) is defined as the ratio of standard deviation to the mean value for each feature. We eliminated those features that were outliers with regards to their coefficient of variance value, specifically those that were one standard deviation away from the mean as the threshold (mean cv=2.05, SD=1.29). This feature selection was applied to the three individual models (1, 2, 3-month models) leading to the final set of 79, 74, and 74 features used by the models. Our feature selection method filtered out noisy features such as linguistic readability of shared Facebook posts, volume of propositions used, and verbs used in posts, prior to model training. Extreme high variance within these features is likely to make the model sensitive to minor distortions thereby leading to poor generalizability. Further, some of the features such as modifying Facebook profile fields and third party app usage had missing values across several participants. This filtering step led to our final sample of 79 features that were used by the best performing 1-month model and are reported in Table 1.

Model selection and parameter tuning: The one-class support vector machine algorithm takes two kinds of parameters as input: 1) the type and parameters for the kernel function and 2) parameter 'nu' that represents an upper bound on the fraction of margin errors and a lower bound of the fraction of support vectors relative to the total number of training examples. We tested multiple kernel types such as rbf, linear, and polynomial. We also tested a range of values for the nu parameter from 0.05 to 0.6. 0.6 was chosen as the upper limit of expected outliers by the model to align with the natural distribution of participants diagnosed with psychotic disorders who had at least one relapse hospitalization in the study. We used grid search for parameter sweeping to identify the best parameters for the one class SVM model.

We applied stratified k-fold (k=5) cross validation to identify the best performing model (for 1, 2, 3 month models). We also considered two and three month relapse periods prior to hospitalization to be incorporated into the 2-month and 3-month models. However, we found poor performance with these temporal periods. A one class SVM model with 2-month periods of relapse and relative health had low sensitivity of 0.21 and the model with 3-month periods of relapse and relative health had low specificity of 0.23.

Ensemble model: We used an ensemble technique based on Boosting that attempts to create a stronger classifier from a number of weak classifiers to build the final model. Boosting approaches are commonly used to lower errors in the combined model and to optimize the advantage and reduce pitfalls of a single model. Through parameter tuning (specifically modifying variance in the nu parameter) multiple configurations of the base model (1-month model) were trained individually as weak learners and three 1-month models were combined using a majority voting mechanism to predict on the unseen test data.

Table 1: Selected features for 1-month model based on the coefficient of variance measure

feature	*cv*	*feature*	*cv*	*feature*	*cv*	*feature*	*cv*
cover photo morning	2.10	exclusive	2.29	indefinite pronoun	1.57	posting morning	1.99
likes morning	1.82	insight	1.91	first person singular	1.59	sadness	3.17
in shares midnight	3.33	second person	1.67	feel	2.76	positive affect	1.46
out shares morning	2.44	past tense	1.42	causation	2.26	posting midnight	2.25
co-tagging midnight	1.62	bio	1.73	posting noon	2.01	total listen	3.08
total in shares	2.65	inhibition	3.29	adverbs	1.47	out shares noon	2.53
out shares midnight	2.65	quantifier	1.91	friending midnight	1.61	third person	1.55
total photo uploads	1.41	conjunction	1.51	future tense	2.74	listen night	3.09
photo upload morning	1.52	present tense	1.10	total likes	1.69	negation	2.33
total out shares	2.53	body	2.21	inclusive	1.56	anger	3.18
photo upload noon	1.29	humans	2.70	discrepancies	1.97	out shares night	2.51
friending morning	1.52	see	1.94	percept	1.40	cover photo midnight	2.19
co-tagging night	2.20	sexual	3.27	photo upload night	1.64	total co-tagging	1.68
listen noon	3.04	health	3.32	listen morning	2.71	tentativeness	1.79
photo upload midnight	1.48	likes noon	1.76	in shares night	2.84	social	0.92
co-tagging noon	2.29	article	1.21	cognitive mechanisms	0.83	auxiliary verbs	1.00
in shares noon	3.00	negative affect	3.09	likes midnight	1.54	achievement	2.29

total friending	1.60	cover photo noon	2.10	work	2.20	likes night	2.04
co-tagging morning	2.23	certainty	2.16	cover photo night	2.01	friending night	1.52
hear	2.99	posting night	1.93	total cover photo	2.07		

References

1) Pitler, E., & Nenkova, A. Revisiting readability: A unified framework for predicting text quality. In Proceedings of the conference on empirical methods in natural language processing. *Association for Computational Linguistics*, 186-195 (2008).
